# Diversity and Distribution of Anaerobic Ammonium Oxidation Bacteria in Hot Springs of Conghua, China

**DOI:** 10.3389/fmicb.2021.739234

**Published:** 2022-01-25

**Authors:** Lan Liu, Ai-Ping Lv, Manik Prabhu Narsing Rao, Yu-Zhen Ming, Nimaichand Salam, Meng-Meng Li, Ze-Tao Liu, Xiao-Tong Zhang, Jing-Yi Zhang, Wen-Dong Xian, Jian-Yu Jiao, Wen-Jun Li

**Affiliations:** ^1^State Key Laboratory of Biocontrol, Guangdong Provincial Key Laboratory of Plant Resources and Southern Marine Science and Engineering Guangdong Laboratory (Zhuhai), School of Life Sciences, Sun Yat-sen University, Guangzhou, China; ^2^State Key Laboratory of Desert and Oasis Ecology, Xinjiang Institute of Ecology and Geography, Chinese Academy of Sciences, Ürümqi, China

**Keywords:** hot springs, anammox bacteria, diversity, physicochemical analysis, putative novel taxa

## Abstract

Anaerobic ammonium oxidation (anammox) is an important process of the nitrogen cycle, and the anammox bacteria have been studied in a wide variety of environments. However, the distribution, diversity, and abundance of anammox bacteria in hot springs remain enigmatic. In this study, the anammox process was firstly investigated in hot springs of Conghua, China. Anammox-like bacterial sequences that closely affiliated to “*Candidatus* Brocadia,” “*Candidatus* Kuenenia,” “*Candidatus* Scalindua,” “*Candidatus* Anammoxoglobus,” and “*Candidatus* Jettenia” were detected. Several operational taxonomic units (OTUs) from this study shared low sequence identities to the 16S rRNA gene of the known anammox bacteria, suggesting that they might be representing putative novel anammox bacteria. A quantitative PCR analysis of anammox-specific 16S rRNA gene confirmed that the abundance of anammox bacteria ranged from 1.60 × 10^4^ to 1.20 × 10^7^ copies L^–1^. Nitrate was a key environmental factor defining the geographical distribution of the anammox bacterial community in the hot spring ecosystem. Dissolved inorganic carbon had a significant influence on anammox bacterial biodiversity. Our findings for the first time revealed that the diverse anammox bacteria, including putative novel anammox bacterial candidates, were present in Conghua hot spring, which extended the existence of anammox bacteria to the hot springs in China and expands our knowledge of the biogeography of anammox bacteria. This work filled up the research lacuna of anammox bacteria in Chinese hot spring habitat and would guide for enrichment strategies of anammox bacteria of Conghua hot springs.

## Introduction

Anaerobic ammonium oxidization (anammox) is a process that can convert ammonium to dinitrogen gas (N_2_) coupled with nitrite reduction under anoxic conditions (Thamdrup, 2012). Anammox is a microbe-mediated process that was predicted in 1977 (Broda, 1977) but was first described in 1995 by Mulder (Mulder et al., 1995) from the bioreactors of wastewater treatment plants. The discovery of anammox challenged the view that heterotrophic denitrification was the only known pathway for nitrogen loss to the atmosphere. The anammox process is mediated by bacteria affiliated to the same monophyletic branch named “*Candidatus* Brocadiales” within the phylum *Planctomyces*. At present, there are 23 *Candidatus* species from 6 different genera of anammox bacteria that have been described, including the *Brocadia anammoxidans*, *Brocadia fulgida*, *Brocadia sinica*, *Brocadia brasiliensis*, *Brocadia caroliniensis*, and *Brocadia sapporoensis* in the genus “*Candidatus* Brocadia” (Strous et al., 1999; Kartal et al., 2008; Hu et al., 2010; Araujo et al., 2011; Narita et al., 2017), *Kuenenia stuttgartiensis* in “*Candidatus* Kuenenia” (Schmid et al., 2000), *Jettenia asiatica*, *Jettenia ecosi*, *Jettenia moscovienalis*, and *Jettenia caeni* in “*Candidatus* Jettenia” (Quan et al., 2008; Ali et al., 2015; Nikolaev et al., 2015; Botchkova et al., 2018), *Scalindua sorokinii*, *Scalindua brodae*, *Scalindua wagneri*, *Scalindua arabica*, *Scalindua sinooilfield*, *Scalindua zhenghei*, *Scalindua profunda*, *Scalindua marina*, and *Scalindua richardsii* in “*Candidatus* Scalindua” (Kuypers et al., 2003; Schmid et al., 2003; Van De Vossenberg et al., 2008; Woebken et al., 2008; Li et al., 2010; Brandsma et al., 2011; Fuchsman et al., 2012; Oshiki et al., 2017; Speth et al., 2017), *Anammoxoglobus propionicus* in “*Candidatus* Anammoxoglobus” (Kartal et al., 2007), and *Brasilis concordiensis* in “*Candidatus* Brasilis” (Viancelli et al., 2011).

Anammox bacteria have a wide distribution in various natural habitats, such as marine (Rich et al., 2008; Dang et al., 2010; Brandsma et al., 2011; Hong et al., 2011), freshwater (Zhang et al., 2007; Yoshinaga et al., 2009; Moore et al., 2011) and terrestrial ecosystems (Humbert et al., 2010; Hu et al., 2011; Zhu et al., 2011), and the animal host (Hoffmann et al., 2009; Mohamed et al., 2010). They were also detected in some special environments, such as high-temperature environments. Using the 16S rRNA gene and *hzo* molecular biomarker, the group of Li (Li et al., 2010) indicated the occurrence of anammox bacteria in nine of seventeen oil reservoirs. The role of anammox in deep-sea hydrothermal vents was investigated by concurrent surveys including the amplification of 16S rRNA gene sequences, ladderanes lipids analysis, and isotope-pairing experiments (Byrne et al., 2009), which strongly indicated the presence and activity of new anammox bacteria in different hydrothermal areas. The existence of anammox bacteria was also detected in the sediments of Bulukey River in Turpan where the surface temperature can reach 75°C in summer (Zhu et al., 2015). As one typical high-temperature environment, there has been limited evidence for the existence and role of anammox bacteria in hot springs (Jaeschke et al., 2009). Therefore, whether anammox bacteria are prevalent in hot springs remains unknown, which impacts our understanding of anammox in the terrestrial nitrogen cycle.

Hot springs, remarkably similar to ancient environments, are the model ecosystem for study on the origin and evolution of life, biogeochemistry, and biogeography (Pace, 1997; Whitaker et al., 2003). The terrestrial hot springs are characterized by extreme geochemical conditions and diverse mineralogical compositions. These geothermal features usually harbor tremendous diversity of uncultivated or unexplored “microbial dark matter” (Jiao et al., 2021b), with novel metabolic capacities and special adaption strategies (Hua et al., 2019; Tan et al., 2019; Luo et al., 2020; Jiao et al., 2021a). Conghua, in the Guangdong province, is a county that encompasses many hot springs. These hot springs are characterized as sodium bicarbonate type containing fluoride, weak radioactive plutonium, and sodium, calcium, potassium, magnesium, silicon dioxide, and other beneficial elements; however, no attention was paid to the microorganisms in it. In this study, six representative hot spring samples with the temperature higher than 45°C were collected from Conghua to assess the diversity and structure of anammox bacteria by Illumina-based 16S rRNA gene amplicon sequencing, measure the abundance of anammox bacteria by quantitative PCR (qPCR) analysis of 16S rRNA gene, and analyze the relations between the physicochemical characteristics of hot spring and the abundance and community structure of anammox bacteria. This study provides a new understanding of the community composition of anammox bacteria in hot springs.

## Materials and Methods

### Sample Collection

Six hot spring samples were collected from three stations: LiuXi River Left (LXRL, 23.649000°N, 113.652000°E), LiuXi River Right (LXRR, 23.649500°N 113.654000°E), and GuanWeiHui (GWH, 23.689363°N, 113.706496°E) of Conghua, Guangdong province in China (Supplementary Figure 1). Origins of five of these samples (GWH1, GWH2, GWH3, LXRR1, and LXRR2) are a subsurface with more than 60 m depth, which limit us to obtain only hot spring water *via* a pump. The source of sample LXRL1 is surface and covered by stones, with the hot spring water constantly gushing out, which made it easier for us to collect hot spring water *via* 10 L white plastic bottles (As One, China). These hot spring water samples were collected during July 2018. Following collection, 20 L of each sample was filtered through 0.22 μm membrane filters (Pall Gelman, Port Washington, NY, United States) with a 150 mm diameter. Biomass on the filter was then transferred to a sterilized 50 mL round-bottom centrifuge tube (Corning, Tewksbury, MA, United States) and stored with dry ice for further DNA extraction. The filtrates were collected in sterilized 250 mL white plastic bottles (As One, China) with 58 mm in diameter and 103 mm in height, transported to the laboratory, and stored at −20°C for subsequent analysis of physicochemical properties.

### Environmental Parameter Analysis

Temperature and pH were measured *in situ* using a portable multimeter (HACH58258; Loveland, CO, United States). Nutrients in the water samples were analyzed according to standard procedures (Lu et al., 2015). The concentrations of total organic carbon, total carbon, and dissolved inorganic carbon were determined using a total organic carbon analyzer (TOC-VCPN; Shimadzu Corp., Tokyo, Japan) based on combustion–oxidation and combustion detection methods. The concentrations of soluble reactive phosphorus, nitrite, and nitrate were measured spectrophotometrically based on molybdenum blue and hydrazine sulfate-NEDD (N-(1-napthyl)ethylenediamine dihydrochloride) methods, respectively. Concentrations of total nitrogen and ammonium were performed using flow injection protocols (WESTCO, SmartChem 200 series flow injection analysis system, Scientific Instruments, Milan, Italy).

### DNA Extraction, PCR Amplification, and Sequencing

The total community DNA from the samples was extracted using the FastDNA Spin Kit (MP Biomedicals, Santa Ana, CA, United States) according to the manufacturer’s protocols. DNA quantity and quality were checked using a NanoDrop^®^ ND-2000 spectrophotometer (Thermo Fisher Scientific, Waltham, MA, United States) and 1.0% agarose-gel electrophoresis. The 16S rRNA gene of anammox bacteria was amplified using a nested PCR approach with first-step primer pair PLA46F (5′-GGATTAGGCATGCAAGTC-3′)/1390R (5′-GACGGGCGGTGTGTACAA-3′) (Schmid et al., 2000) and second-step primer pair Amx368F (5′-TTCGCAATGCCCGAAAGG-3′)/Amx820R (5′-AAAACCCCTCTACTTAGTGCCC-3′) (Wang et al., 2017). PCR reaction conditions were as follows: 94°C for 4 min; 32 cycles of 94°C for 30 s, 56°C (first step) or 58°C (second step) for 30 s, followed by 72°C for 1 min; and a final 10 min extension at 72°C. High-throughput paired-end Illumina MiSeq (Illumina, San Diego, CA, United States) sequencing (2 × 300 bp) was performed at Genewiz, Suzhou, China.

### Quantitative PCR Analysis

The abundance of the 16S rRNA gene of anammox bacteria was determined in triplicate on a Bio-Rad iQ5 thermal cycler (Bio-Rad Laboratories) using primer sets AMX-808F (5′-ARCYGTAAACGATGGGCACTAA-3′)/AMX1040R (5′-CAGCCATGCAACACCTGTRATA-3′) (Hamersley et al., 2007) with a thermal profile of 3 min at 95°C, followed by 40 cycles of 30 s at 95°C, 30 s at 55°C, and 30 s at 72°C. SYBR Green I-based real-time PCR assays were carried out in a volume of 20 μL, containing 10 μL of SYBR Premix Ex Taq (TAKARA, Guangzhou, China), 0.2 μL of each primer, and 1 μL DNA template, and was topped up with ddH_2_O to a total volume of 20 μL. A standard plasmid carrying 16S rRNA gene fragment was generated by amplifying them from the extracted DNA sample and cloning into pEASY-T1 Vector (TransGen, Beijing, China). The concentration of plasmid DNA was determined on a NanoDrop^®^ ND-2000 spectrophotometer (Thermo Fisher Scientific, Waltham, MA), and the copy number of the target gene was calculated directly from the concentration of the extracted plasmid DNA. A significant linear relationship of 16S rRNA gene amplification (*R*^2^ = 0.9966) was obtained between the log 10 values of the standard plasmid DNA concentration (2.00 × 10–2.00 × 10^7^ copies L^–1^) and the associated threshold cycles (Ct). Melting curves and gel electrophoresis were performed to confirm the specificity of the qPCR amplification. In all experiments, blank controls containing no template DNA were performed under the same qPCR procedure to ensure no contamination in our PCR system and the reliability of the results.

### Data Analysis

Analysis of raw Illumina fastq files was performed using Quantitative Insights Into Microbial Ecology 2 (QIIME 2, ver. 2019.1) (Bolyen et al., 2019). DADA 2 (Callahan et al., 2016) was used to filter, trim, denoise, and merge sequences. Chimeric sequences were identified and removed. Taxonomy was assigned to chimeric-free sequences using a q2-feature classifier (Bokulich et al., 2018) classify-sklearn naïve Bayes taxonomy classifier against the Silva 132 97% OTU reference sequences (Quast et al., 2013). Phylogenetic analysis of the representative 16S rRNA gene sequences of anammox bacteria was conducted with the MEGA 7.0 software, and the phylogenetic tree was visualized and annotated using iTOL (Letunic and Bork, 2019). Optimal growth temperatures of top 30 dominant OTUs and the reference targets from the NCBI database were calculated by using the GC contents (Kimura et al., 2006). To analyze the alpha diversity, Shannon (Shannon--Weaver), Simpson (Gini--Simpson), and Pielou indices were calculated by using the R program (v.3.5.3)^1^ with the vegan package. UpSetR (Conway et al., 2017) was used to investigate whether exclusively shared OTUs existed among the samples. The relationships of anammox bacterial diversity, anammox bacterial abundance, and physicochemical parameters of samples were calculated by Spearman’s correlation analysis using R (corrplot package). The contribution of the chemical characteristics of the six samples to the variances of anammox bacterial communities was assessed with variance partitioning analysis and canonical correspondence analysis (CCA) using the package vegan^2^ of the R program (v.3.5.3; see text footnote 1).

## Results

### Physicochemical Analysis of the Sampling Sites

The basic physicochemical properties of hot spring samples collected from Conghua are shown in Table 1. There was little change in pH (6.5–7.5) and temperature (49–58°C) among all of the sites. A high degree of heterogeneity in concentrations of total organic carbon, total nitrogen, and ammonium was observed in the examined samples, which varied within the range of 0.054–14.230, 4.560–25.220, and 0.005–0.271 mg/L, respectively. All samples had high concentrations of total carbon and dissolved inorganic carbon with values ranging from 21.250 to 36.050 mg/L and from 16.940 to 28.560 mg/L. However, concentrations of nitrate (0.020–0.113 mg/L), nitrite (0.002–0.030 mg/L), and soluble reactive phosphorus (0–0.350 mg/L) were relatively low at all sites.

**TABLE 1:** Physicochemical characteristics of the samples collected from the hot springs of Conghua, China.

Samples	Temperature (°C)	pH	TOC (mg/L)	TC (mg/L)	DIC (mg/L)	TN (mg/L)	NH_4_^+^ (mg/L)	P (mg/L)	NO_3_^–^ (mg/L)	NO_2_^–^ (mg/L)
GWH1	49	7.20	0.054	21.250	21.190	25.220	0.261	0.000	0.020	0.002
GWH2	54	6.50	0.576	28.140	27.560	17.460	0.271	0.002	0.036	0.004
GWH3	52	7.00	1.294	29.030	27.740	4.560	0.046	0.000	0.036	0.005
LXRL1	58	6.50	14.23	31.170	16.940	23.990	0.121	0.004	0.027	0.030
LXRR1	55	7.50	1.269	28.680	27.410	22.190	0.100	0.035	0.113	0.010
LXRR2	54.5	7.50	7.488	36.050	28.560	21.290	0.005	0.004	0.103	0.003

*TOC, total organic carbon; TC, total carbon; DIC, dissolved inorganic carbon; TN, total nitrogen; P, soluble reactive phosphorus; NH_4_^+^, ammonia; NO_3_^–^, nitrate; NO_2_^–^, nitrite.*

### Diversity of Anammox Bacteria

**FIGURE 1:**
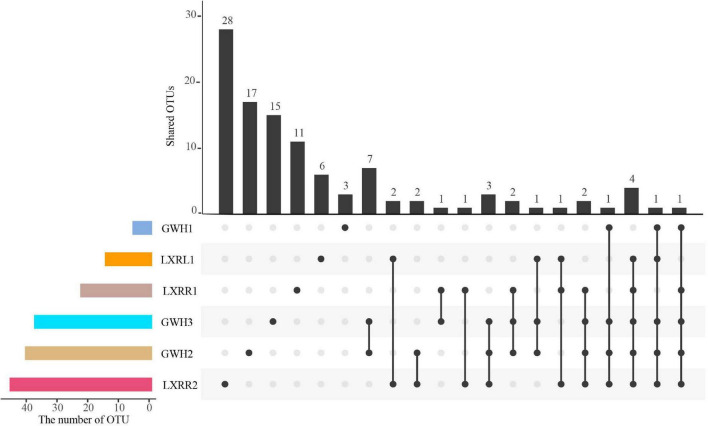
An UpSetR plot showing the shared and unique OTUs at 97% similarity among the samples. UpSetR is an R package; it employs a matrix-based layout to show intersections of sets and their sizes, and it is implemented using ggplot2 and allows data analysts to easily generate UpSet plots for their own data. The strip at the bottom left shows the number of OTUs included in each sample. The dot and line at the bottom right represent the subsets of samples. The number of relevant OTUs in each subset is represented in the histogram, which is the upper part of the whole plot.

After quality control, a total of 181,802 high-quality anammox bacterial 16S rRNA gene sequences were obtained ranging between 13,884 and 57,231 sequences per sample (Table 2), which were clustered into 109 operational taxonomic units (OTUs) based on the 97% similarity level. The numbers of anammox bacterial OTUs in GWH1, GWH2, GWH3, LXRL1, LXRR1, and LXRR2 were 6, 41, 37, 15, 23, and 46, respectively. Each sample had its own unique anammox bacterial OTUs, with the proportion of independent anammox bacterial OTUs in samples GWH1, GWH2, GWH3, LXRL1, LXRR1, and LXRR2 of 50.0, 41.5, 40.5, 40.0, 47.8, and 60.9%, respectively, however, no OTUs were shared by all the samples (Figure 1). The alpha diversity of anammox bacteria was estimated by calculating the Shannon, Simpson, and Pielou indices. The Shannon and Simpson indices showed that the highest diversity of anammox bacteria were detected in sample GWH3 (4.50 and 0.94, respectively), whereas sample GWH1 (1.13) and sample LXRR1 (0.40) had the lowest diversity based on the Shannon index and Simpson index, respectively. The Pielou of anammox bacteria ranged from 0.35 to 0.86. Rarefaction curves for the anammox bacterial 16S rRNA gene at 97% similarity showed that the high-throughput sequencing could supply enough bio-information to investigate the community composition and diversity of anammox bacteria in the current study.

**TABLE 2:** Diversity characteristics and abundance of anammox bacterial 16S rRNA gene.

Sample	High-quality	OTUs	Shannon	Simpson	Pielou	Abundance
	sequences					(copies L^−1^)
GWH1	31,266	6	1.13	0.45	0.44	1.20 × 10^7^
GWH2	23,742	41	2.84	0.69	0.53	2.30 × 10^5^
GWH3	13,884	37	4.5	0.94	0.86	8.51 × 10^5^
LXRL1	28,754	15	2.34	0.74	0.6	1.60 × 10^4^
LXRR1	57,231	23	1.6	0.4	0.35	5.55 × 10^4^
LXRR2	26,652	46	4.48	0.91	0.81	2.26 × 10^5^

### Community Composition of Anammox Bacteria

**FIGURE 2:**
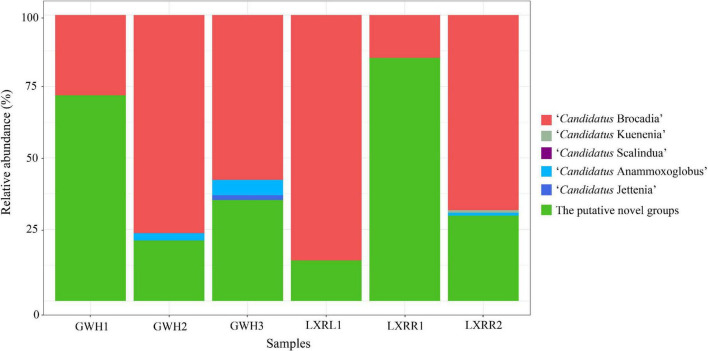
Taxonomic classifications of anammox 16S rRNA gene sequences retrieved from the hot spring at the genus level. The colors indicate different taxa. The horizontal axis is the sample, and the vertical axis is the relative abundance.

Based on the phylogenetic analysis, anammox-like bacteria that were closely affiliated to five known anammox bacterial genera were detected in the hot springs of Conghua. Among the 109 anammox bacterial OTUs in the collected samples, 65 OTUs were affiliated with “*Candidatus* Brocadia” (47.53%), “*Candidatus* Kuenenia” (0.134%), “*Candidatus* Anammoxoglobus” (0.896%), “*Candidatus* Jettenia” (0.133%), and “*Candidatus* Scalindua” (0.005%). The remaining unknown anammox bacterial OTUs accounted for 51.302%, which indicated that many putative novel anammox bacteria existed in the hot spring of Conghua. “*Candidatus* Brocadia” was the dominant genus in samples GWH2, GWH3, LXRL1, and LXRR2, while the putative novel anammox bacteria were dominant in samples GWH1 and LXRR1. “*Candidatus* Jettenia” was detected in GWH2, GWH3, and LXRR2; however, “*Candidatus* Kuenenia” and “*Candidatus* Scalindua” were only observed in LXRR2 and GWH3, respectively. The community structure of anammox bacteria in hot springs at the genus level is depicted in Figure 2.

**FIGURE 3:**
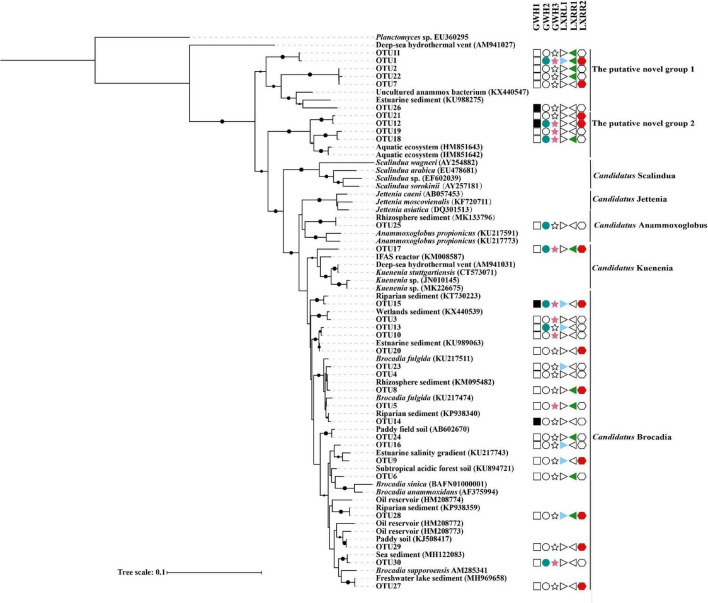
The Neighbor-Joining tree showing the relationship among the top 30 dominant OTU sequences and the reference sequences from the NCBI database. Different shapes with different colors were used to code 6 samples, and empty symbols represent no sequence in the corresponding sample. Bootstrap values were 1,000 replicates. The scale bar represented 10% of sequence divergence.

The anammox bacterial distribution pattern was verified at the OTU level. A phylogenetic tree (Figure 3) was constructed to exhibit relationships between the representative sequences of the top 30 dominant OTUs and anammox bacterial reference sequences from the NCBI database. The result showed that most OTUs were widely distributed in the six samples. Seven phylogenetic clades were shown in the phylogenetic tree with five clades containing dominant OTUs. In *Brocadia* clade, eighteen OTUs were retrieved from the samples. OTU 17 and OTU 25 were assigned to the “*Candidatus* Kuenenia” and “*Candidatus* Anammoxoglobus” clades, respectively, and ten OTUs belonged to the putative novel anammox genera, which means that genes of “*Candidatus* Brocadia” had the highest diversity, and “*Candidatus* Kuenenia” and “*Candidatus* Anammoxoglobus” had a lower diversity. BLASTN analyses revealed that the retrieved anammox bacterial 16S rRNA gene sequences in the hot spring of Conghua were also closely affiliated with sequences detected in paddy field soil, aquatic ecosystem, reactor, and sediment from the rhizosphere, wetland, riparian, estuarine, freshwater lake, and sea with a 92.65–100% sequence similarity. Supplementary Table 1 shows that the optimal growth temperatures of most targets including the sequences from petroleum reservoirs and hydrothermal vents were below 35°C.

### The Abundance of Anammox Bacteria

Anammox bacterial 16S rRNA genes were used to estimate the abundance of anammox bacteria by the qPCR method. In this study, melting curve analyses confirmed that fluorescent signals were derived from the specific PCR products during qPCR quantification. The qPCR results with 97.43% amplification efficiency showed that the abundance of anammox bacteria ranged from 1.60 × 10^4^ to 1.20 × 10^7^ copies L^–1^ (Table 2). Sample GWH1 (1.20 × 10^7^ copies L^–1^) had the highest abundance of anammox bacteria, followed by sample GWH3 (8.51 × 10^5^ copies L^–1^). The lowest abundance of anammox bacteria was observed in sample LXRL1 (1.60 × 10^4^ copies L^–1^).

### Effects of Environmental Factors on Microbial Communities

**FIGURE 4:**
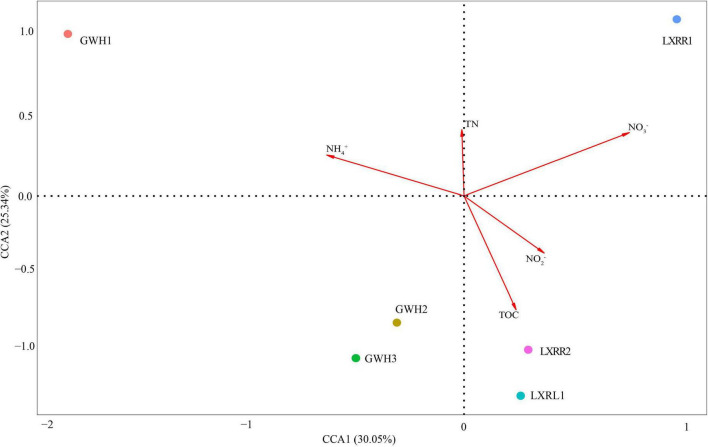
Canonical correspondence analysis (CCA) ordination plots for the first two principal dimensions to show the relationship between anammox bacterial community composition and physicochemical parameters. The percentage indicates the interpretation of axis to the diversification of anammox bacterial community. Correlations between environmental variables and CCA axes are represented by the length and angle of arrows. Circles with different colors represent diverse samples. Arrow length expresses relative importance of an environmental variable; the longer the arrow, the greater influence of environmental factors. Dropping perpendicular to the arrows from each of the samples indicates the strength of the environmental factor’s influence on the sample; the shorter distance, the stronger the effect of the environmental factor on the sample. The sample is located in the same direction as the arrow, indicating that the environmental factor is positively correlated with the change of the bacterial community, and the sample is located in the opposite direction of the arrow, indicating that the environmental factor is negatively related to the change of the bacterial community. TOC and TN mean total organic carbon and total nitrogen, respectively.

CCA was conducted to show the potential relationships between environmental variables and the microbial communities of anammox bacteria in hot spring samples (Figure 4). The environmental factors in the first two CCA axes, respectively, explained 30.05 and 25.34% of the total variance for anammox bacterial OTU composition. Results showed that total organic carbon, ammonium, and nitrate were found to be the main factors affecting the distribution of anammox bacteria in Conghua hot spring.

**FIGURE 5:**
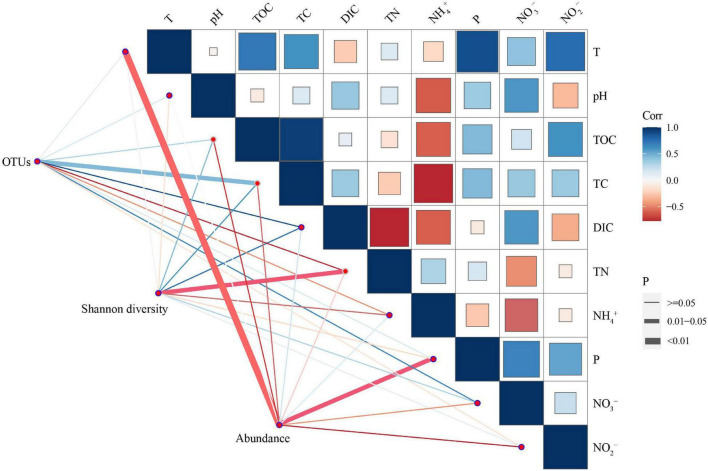
Spearman’s correlation analysis based on the 16S rRNA gene of anammox bacterial diversity, abundance, and physicochemical parameters (including T, pH, TOC, TC, DIC, TN, NH_4_^+^, P, NO_2_^–^, and NO_3_^–^) in six hot spring samples. The color scale denotes the nature of the correlation, with blue and red denoting the positive and negative correlations, respectively. The line thickness denotes different *p*-values; the thicker the line, the more significant correlation. On the contrary, the thinner the line, the less significant the correlation. T, temperature; TOC, total organic carbon; TC, total carbon; DIC, dissolved inorganic carbon; TN, total nitrogen; P, soluble reactive phosphorus; NH_4_^+^, ammonia; NO_3_^–^, nitrate; NO_2_^–^, nitrite.

Spearman’s correlation analysis was used to illustrate the correlations among anammox gene abundances, diversity, and environmental factors. The results indicated that anammox bacterial abundance was negatively correlated with temperature (*p* < 0.01) and soluble reactive phosphorus (*p* < 0.05) (Table 3). Shannon diversity was negatively correlated with total nitrogen (*p* < 0.05). Anammox bacterial OTUs illustrated significantly positive correlations with dissolved inorganic carbon (*p* < 0.05). In addition, total organic carbon had a highly significant positive correlation with total carbon (*p* < 0.01), and soluble reactive phosphorus had a significantly positive correlation with temperature (*p* < 0.05) (Figure 5).

**TABLE 3:** Spearman rank correlation analysis of environmental factors with the abundance and diversity of anammox bacterial community in Conghua hot spring.

	Temperature	pH	TOC	TC	DIC	TN	NH_4_^+^	P	NO_3_^–^	NO_2_^–^	Abundance	Shannon diversity	OTUs
Temperature	1.00	–0.03	0.71	0.60	–0.26	0.14	–0.20	0.88*	0.41	0.77	−1.00**	–0.03	0.09
pH		1.00	–0.09	0.15	0.38	0.15	–0.62	0.36	0.58	–0.32	0.03	–0.15	0.15
TOC			1.00	0.94**	0.09	–0.14	–0.60	0.44	0.20	0.60	–0.71	0.49	0.26
TC				1.00	0.37	–0.26	–0.77	0.44	0.38	0.37	–0.60	0.60	0.49
IC					1.00	–0.77	–0.60	–0.09	0.58	–0.37	0.26	0.77	0.89*
TN						1.00	0.31	0.18	–0.46	–0.09	–0.14	−0.89*	–0.77
NH_4_^+^							1.00	–0.26	–0.55	–0.09	0.20	–0.54	–0.43
P								1.00	0.67	0.53	−0.88*	–0.18	0.18
NO_3_^–^									1.00	0.23	–0.41	0.32	0.64
NO_2_^–^										1.00	–0.77	0.09	–0.14
Abundance											1.00	0.03	–0.09
Shannon diversity												1.00	0.77
OTUs													1.00

Positive numbers indicate a positive correlation, and negative numbers indicate a negative correlation. “*” denotes a p-value of < 0.05, and “**” denotes a p-value of < 0.01, the lower p-value, the more significant correlation.

*TOC, total organic carbon; TC, total carbon; DIC, resolved inorganic carbon; TN, total nitrogen; P, soluble reactive phosphorus; NH_4_^+^, ammonia; NO_3_^–^, nitrate; NO_2_^–^, nitrite.*

## Discussion

This study was the first time to investigate the diversity of anammox bacteria and to quantify the number of anammox 16S rRNA genes in hot springs of China. The influences of environmental factors on the distribution and diversity of anammox bacterial communities were also studied. Our data indicated that anammox bacteria can thrive in the hot spring of China, including putative novel taxa, which may imply that anammox bacteria could play a significant role in the nitrogen cycle of hot spring environments and a more in-depth research is necessary. Considering the detection of anammox bacteria from hot springs of California and Nevada (Jaeschke et al., 2009), we speculated that anammox bacteria might be widely distributed in the hot spring ecosystem. As expected, we reported the presence of anammox bacteria in Chinese hot springs during this study, which expanded our knowledge of the biogeography of anammox bacteria. Our study illustrated a high biodiversity of anammox bacteria in the hot spring of Conghua with five known genera of anammox bacteria (“*Candidatus* Scalindua,” “*Candidatus* Brocadia,” “*Candidatus* Kuenenia,” “*Candidatus* Jettenia,” and “*Candidatus* Anammoxoglobus”), and “*Candidatus* Brocadia” was identified as the dominant genus. To date, the diversity of anammox bacteria has been studied in various ecosystems. A low diversity of anammox bacteria was reported in marine ecosystems, which was mainly restricted to “*Candidatus* Scalindua” (Schmid et al., 2007). Anammox bacteria diversity in the freshwater ecosystem was higher than in the marine ecosystem; for example, the *hzsB* gene sequences were closely affiliated to “*Candidatus* Kuenenia” and “*Candidatus* Brocadia” in some freshwater extreme environments (Zhu et al., 2015). Anammox diversity in the terrestrial systems was higher in soil than in freshwater environments; for example, in wetland and dryland soils, three different genera of anammox bacteria were detected, including “*Candidatus* Kuenenia,” “*Candidatus* Brocadia,” and “*Candidatus* Jettenia” (Zhao et al., 2018). In acidic red soils, anammox bacteria were related to “*Candidatus* Brocadia,” “*Candidatus* Scalindua,” “*Candidatus* Kuenenia,” and “*Candidatus* Anammoxoglobus” (Wu et al., 2018). The above discussions indicated that in natural ecosystems, the highest diversity of anammox bacteria occurred in terrestrial systems, followed by freshwater systems and marine systems, which was in accordance with our finding that anammox bacteria had a higher diversity in hot springs. Our finding was also consistent with the understanding that “*Candidatus* Kuenenia,” “*Candidatus* Brocadia,” “*Candidatus* Anammoxoglobus,” and “*Candidatus* Jettenia” co-occurred in terrestrial environments (Gao et al., 2018). Furthermore, the microbial diversity of anammox bacteria exhibited a negatively significant correlation with total nitrogen, which was inconsistent with previous results that a higher total nitrogen concentration may provide an environment favorable for the distribution and growth of anammox bacteria (Shen et al., 2016).

The community composition of anammox bacteria in the six samples was different. The genera “*Candidatus* Brocadia,” “*Candidatus* Scalindua,” “*Candidatus* Anammoxoglobus,” and “*Candidatus* Jettenia” were detected in GWH2, “*Candidatus* Brocadia,” “*Candidatus* Anammoxoglobus,” and “*Candidatus* Jettenia” were present in GWH3, and “*Candidatus* Brocadia,” “*Candidatus* Anammoxoglobus,” and “*Candidatus* Kuenenia” were observed in LXRR2. Among all the known anammox bacteria, “*Candidatus* Brocadia” was found in all the six samples; this finding might be related to the diverse metabolic pathways reported to “*Candidatus* Brocadia” (Gori et al., 2011), making it the most common anammox genus in terrestrial ecosystems, including the hot spring ecosystem. Interestingly, “*Candidatus* Brasilis” was found in 2011 (Viancelli et al., 2011), and it included only one *Candidatus* species to date. However, since 2011, there was no report about it, and among the six known anammox bacteria, “*Candidatus* Brasilis” was not detected in this study, which might be due to that “*Candidatus* Brasilis” was not widely distributed, or the primers might not suitable for “*Candidatus* Brasilis.”

So far, there are 23 *Candidatus* species from six different genera of anammox bacteria that have been discovered from different environments. There are still a large number of anammox bacteria waiting to be excavated. For example, the putative novel anammox bacteria in surface sediment from the Dongjiang River through the Pearl River Estuary to the South China Sea accounted for 3.79% (Li et al., 2020). Similarly, the putative novel anammox bacteria accounted for 6.83% in the acidic red soils of southern China (Wu et al., 2018), and the proportion of putative novel anammox bacteria in sediment cores of the Pearl River Estuary was 45.12% (Wu et al., 2020). The putative novel anammox bacteria were also detected in freshwater wetlands of southeastern China (Shen et al., 2016) and Dongjiang River (Sun et al., 2014a). In our study, the putative novel anammox bacteria were up to 51.30%, which was higher than in the previous studies (Wu et al., 2018, 2020; Li et al., 2020). Additionally, two new clusters of putative anammox bacterial 16S rRNA genes were formed in the phylogenetic tree (Figure 3). Sequences of OTU1, OTU2, OTU7, OTU11, OTU22, and OTU26 showed 92.65–94.53% identity to the 16S rRNA genes of known anammox bacteria from estuarine sediments of southeastern China, and sequences of OTU12, OTU18, OTU19, OTU21, and OTU26 showed 96.12–96.77% identity to the 16S rRNA gene of anammox bacteria from aquatic ecosystems (Hirsch et al., 2011). It is worth noting that the predicted optimal growth temperatures of OTU1, OTU2, OTU7, OTU11, and OTU22 were ranging from 47.90 to 50.37°C (Supplementary Table 1). These results revealed that hot springs harbored a large number of novel anammox bacteria, indicating that anammox bacteria in the hot spring of Conghua were worthy for an in-deep mining. Spearman’s rank correlation analysis showed that dissolved inorganic carbon had a significant positive correlation with OTUs, which was consistent with previous results that anammox bacteria are chemolithoautotrophic microorganisms (Strous et al., 2006; Jetten et al., 2010). This implied that dissolved inorganic carbon was the key environmental factor shaping the distribution of anammox bacteria in the hot spring of Conghua. Interestingly, no significant correlation was observed between the OTUs and diversity, indicating that each sample had its own unique dominant group, which was further supported by the results from UpSetR analysis.

Anammox bacteria were active at 6–43°C with an optimal temperature of 35°C in laboratory bioreactors. However, anammox bacteria were also observed at 52°C in hot spring (Jaeschke et al., 2009), 72°C in petroleum reservoirs (Li et al., 2010), 75°C in freshwater (Zhu et al., 2015), and even 60–80°C in hydrothermal vents (Russ et al., 2013). The dominant anammox species in these high-temperature habitats were either *Brocadia* or *Kuenenia*. In our study, the samples have a temperature of up to 58°C with *Brocadia* as a dominant anammox species, which is consistent with the findings that some anammox species can endure high temperatures. This result indicated that anammox bacteria were not only mesophilic but also thermostable. The special ecophysiological feature of anammox bacteria should be attributed to their specific cellular structure. To maintain the constant membrane fluidity at different temperatures, anammox bacteria could modify their membrane composition to adapt to the change of temperature (Jaeschke et al., 2009). Overall, the temperature may not be the limiting factor for the occurrence of anammox bacteria, and anammox can occur in a wide temperature range. However, the temperature tolerance and the optimal temperature in high-temperature habitats need to be studied in future research.

Among the anammox bacteria analysis, many anammox bacterial DNAs were collected from the sediments or soil of different ecosystems. The abundance of anammox bacteria was measured by the number of gene copies per gram of soil. For example, the global distribution of anaerobic ammonia oxidation bacteria in wetland, dryland, and groundwater aquifer revealed by the *hzsB* gene showed that the gene abundance of anammox bacteria in wetlands was 5.42 × 10^4^−9.56 × 10^6^ copies/g of dry soil, sediments from freshwater rivers at 6.9 × 10^4^–8.9 × 10^5^ copies/g of dry soil, and freshwater lakes at 5.4 × 10^4^–9.6 × 10^6^ copies/g of dry soil, and that in groundwater aquifers was high, ranging from 5.93 × 10^5^ to 9.12 × 10^6^ copies/g of dry soil (Wang et al., 2019). However, some anammox bacterial DNA was extracted from the water, and the abundance of anammox bacteria was measured by the number of cells per milliliter sample (Byrne et al., 2009; Li et al., 2010; Sun et al., 2014a). In our study, the abundance of anammox bacteria ranged from 1.60 × 10^4^ to 1.20 × 10^7^ copies L^–1^. Based on the estimation of 3.6 copy numbers of 16S rRNA gene per bacterial cell genome (Harms et al., 2003), the anammox bacterial abundance was calculated to be 4.40–3.3 × 10^3^ cells mL^–1^. The overall abundance of anammox bacteria observed in this study was lower than the values reported for most deep-sea sediments (Schmid et al., 2007), and high-temperature oil reservoirs (Li et al., 2010), but almost had the same range as reported in Dongjiang River (Sun et al., 2014a). Temperature and soluble reactive phosphorus were negatively correlated with anammox abundance, suggesting that higher temperature and soluble reactive phosphorus concentration could restrain the growth of anammox bacteria.

Previous studies have shown that many different environmental factors can affect anammox bacterial diversity and distribution. For example, temperature was a key environmental factor shaping the distribution and diversity of anammox bacteria in the coastal estuaries of China (Yang et al., 2017). Organic carbon influenced the distribution of anammox bacteria in Qiantang River sediments (Hu et al., 2012) and Yangtze estuary marsh sediment (Hou et al., 2013). Ammonium and nitrite amendments would change the community compositions of anammox bacteria in mangrove sediments to ammonium or nitrite (Li and Gu, 2013), and salinity was one of the key factors driving the biogeography of anammox bacteria (Sonthiphand et al., 2014). This selection is owing to the physiological properties of anammox bacteria, such as the optimum growth temperature and pH, and the affinity for ammonia and nitrite (Oshiki et al., 2011). In the present study, a CCA test was conducted to find the potential relationship between the distribution of anammox bacteria and the environmental factors as the steepest gradient of detruded correspondence analysis was higher than 4.0, and the result of CCA suggested that ammonium and total organic carbon were the main factors affecting the distribution of anammox bacteria in hot spring of Conghua, Additionally, nitrate content had a significant contribution to anammox bacterial community structure, which may be attributed to an increased supply of nitrite *via* reduction of nitrate, and have also been observed in Dongjiang River (Sun et al., 2014b). However, the role of other unidentified ecological parameters except for the present factors also cannot be ruled out.

## Conclusion

Evidence for the occurrence of anammox in this study was demonstrated by the amplification of 16S rRNA gene sequences and quantitative PCR analysis, suggesting that anammox bacteria were present in the hot springs of Conghua. Phylogenetic analysis of the 16S rRNA gene sequences showed a higher diversity of anammox bacteria in hot springs of Conghua than other high-temperature habitats, such as deep-sea hydrothermal vent, hot spring, and the petroleum reservoir. The anammox community was dominated by “*Candidatus* Brocadia” and harbored putative novel anammox bacterial candidates. Nitrate played a key environmental factor in regulating the distribution of the anammox bacterial community in Conghua hot springs. These results extend our understanding of the community dynamics, environmental importance, and biogeography of anammox bacteria in hot spring ecosystems and would guide for future enrichment strategies of anammox bacteria in Conghua hot springs.

## Data Availability Statement

The datasets presented in this study can be found in online repositories. The names of the repository/repositories and accession number(s) can be found below: NCBI SRA BioProject, accession no: PRJNA751159; National Omics Data Encyclopedia, accession no: OEP002743.

## Author Contributions

W-JL, LL, and J-YJ jointly conceived the study. W-DX, Z-TL, and J-YZ performed the sample collection. LL designed the experiments. A-PL, Y-ZM, and M-ML did the experiments. LL, X-TZ, and J-YJ analyzed the culture-independent data. LL, J-YJ, MPNR, NS, and W-JL wrote the manuscript. W-JL gave the final approved of the manuscript to be published. All authors read and approved the final manuscript.

## Conflict of Interest

The authors declare that the research was conducted in the absence of any commercial or financial relationships that could be construed as a potential conflict of interest.

## Publisher’s Note

All claims expressed in this article are solely those of the authors and do not necessarily represent those of their affiliated organizations, or those of the publisher, the editors and the reviewers. Any product that may be evaluated in this article, or claim that may be made by its manufacturer, is not guaranteed or endorsed by the publisher.

## Footnotes

1
http://www.r-project.org/

2
https://CRAN.R-project.org/package=vegan
