# Supplementary material for: Diversity and Distribution of Anaerobic Ammonium Oxidation Bacteria in Hot Springs of Conghua, China
Source: Front Microbiol. 2022 Jan 25;12:739234. doi: 10.3389/fmicb.2021.739234 (PMC8822059; doi:10.3389/fmicb.2021.739234)
Supplement: Supplementary file 1 [file Data_Sheet_1.docx]

Supplementary Material

**Diversity and Distribution of Anaerobic Ammonium Oxidation Bacteria**

**in Hot Springs of Conghua, China**

**Lan Liu^1^, Ai-Ping Lv^1^,** **Manik Prabhu Narsing Rao^1^, Yu-Zhen Ming^1^,** **Nimaichand Salam^1^, Meng-Meng Li^1^,** **Ze-Tao Liu^1^, Xiao-Tong Zhang^1^, Jing-Yi Zhang^1^, Wen-Dong Xian^1^, Jian-Yu Jiao^1^*, Wen-Jun Li^1,2^***

^1^State Key Laboratory of Biocontrol, Guangdong Provincial Key Laboratory of Plant Resources and Southern Marine Science and Engineering Guangdong Laboratory (Zhuhai), School of Life Sciences, Sun Yat-Sen University, Guangzhou, 510275, PR China

^2^State Key Laboratory of Desert and Oasis Ecology, Xinjiang Institute of Ecology and Geography, Chinese Academy of Sciences, Urumqi, 830011, PR China

*** Correspondence:**

Wen-Jun Li, Sun Yat-Sen University, Guangzhou, 510275, PR China

Email: liwenjun3@mail.sysu.edu.cn

Jian-Yu Jiao, Sun Yat-Sen University, Guangzhou, 510275, PR China

Email: jiaojy5@mail.sysu.edu.cn

**Figure S1. Sampling location.**

**Table.S1 The optimal growth temperature of top 30 dominant OTU sequences in this study and the reference sequences from the NCBI database including the sequences from petroleum reservoirs and hydrothermal vents.**

| **Clades** | **Targets** | **Optimal growth temperature (**ºC**)^a^** | **Source** |
| --- | --- | --- | --- |
| Reference target | Deep-sea hydrothermal vents (AM941027) | 40.03 | Deep-sea hydrothermal vents |
| The putative novel group 1 | OTU11 | 46.66 | Hot spring (In this study) |
|  | OTU1 | 47.90 | Hot spring (In this study) |
|  | OTU2 | 50.37 | Hot spring (In this study) |
|  | OTU22 | 50.37 | Hot spring (In this study) |
|  | OTU7 | 49.13 | Hot spring (In this study) |
|  | Uncultured anammox bacterium (KX440547) | 38.01 | Wetlands |
|  | Estuarine sediment (KU988275) | 41.87 | Estuarine sediment |
|  | OTU26 | 35.67 | Hot spring (In this study) |
| The putative novel group 2 | OTU21 | 8.97 | Hot spring (In this study) |
|  | OTU12 | 10.20 | Hot spring (In this study) |
|  | OTU19 | 21.30 | Hot spring (In this study) |
|  | OTU18 | 22.53 | Hot spring (In this study) |
|  | Aquatic ecosystem (HM851643) | 14.76 | Groundwater |
|  | Aquatic ecosystem (HM851642) | 17.29 | Groundwater |
| *Candidatus* Scalindua | *Scalindua wagneri* (AY254882) | 9.88 | Reactor |
|  | *Scalindua* *arabica* (EU478681) | 10.47 | Marine |
|  | *Scalindua* sp. (EF602039) | 11.39 | Marine |
|  | *Scalindua* *sorokinii* (AY257181) | 4.05 | Marine |
| *Candidatus* Jettenia | *Jettenia* *caeni* (AB057453) | 34.62 | Reactor |
|  | *Jettenia* *moscovienalis* (KF720711) | 25.06 | Reactor |
|  | *Jettenia* *asiatica* (DQ301513) | 31.70 | Reactor |
| *Candidatus* Anammoxoglobus | Rhizosphere sediment (MK133796) | 4.05 | Rhizosphere sediment |
|  | OTU25 | 32.29 | Hot spring (In this study) |
|  | *Anammoxoglobus* *propionicus* (KU217773) | 34.86 | Estuary sediment |
|  | *Anammoxoglobus* *propionicus* (KU217591) | 34.86 | Estuary sediment |
| *Candidatus* Kuenenia | OTU17 | 30.03 | Hot spring (In this study) |
|  | IFAS reactor (KM008587) | 32.96 | Reactor |
|  | Deep-sea hydrothermal vent (AM941031) | 26.53 | Deep-sea hydrothermal vent |
|  | *Kuenenia* *stuttgartiensis* (CT573071) | 26.92 | Marine |
|  | *Kuenenia* sp. (MK226675) | 34.86 | River |
|  | *Kuenenia* sp. (JN010145) | 30.95 | Tidallflat sediment |
| *Candidatus* Brocadia | Riparian sediment (KT730223) | 37.33 | Riparian sediment |
|  | OTU15 | 37.33 | Hot spring (In this study) |
|  | Wetlands sediment (KX440539) | 27.46 | Wetlands sediment |
|  | OTU3 | 27.46 | Hot spring (In this study) |
|  | OTU13 | 27.46 | Hot spring (In this study) |
|  | OTU10 | 28.70 | Hot spring (In this study) |
|  | Estuarine sediment (KU989063) | 29.93 | Estuarine sediment |
|  | OTU20 | 29.93 | Hot spring (In this study) |
|  | *Brocadia* *fulgida* (KU217511) | 26.23 | Estuarine sediment |
|  | OTU23 | 25.00 | Hot spring (In this study) |
|  | OTU4 | 26.23 | Hot spring (In this study) |
| *Candidatus* Brocadia | Rhizosphere sediment (KM095482) | 18.55 | Rhizosphere sediment |
|  | OTU8 | 27.46 | Hot spring (In this study) |
|  | *Brocadia* *fulgida* (KU217474) | 27.46 | Estuary sediment |
|  | OTU5 | 25.00 | Hot spring (In this study) |
|  | Riparian sediment (KP938340) | 26.23 | Riparian sediment |
|  | OTU14 | 25.00 | Hot spring (In this study) |
|  | Paddy field soil (AB602670) | 37.17 | Soil |
|  | OTU24 | 33.63 | Hot spring (In this study) |
|  | OTU16 | 38.56 | Hot spring (In this study) |
|  | Estuarine sediment (KU217743) | 39.80 | Estuary sediment |
|  | OTU9 | 37.33 | Hot spring (In this study) |
|  | Subtropical acidic forest soil (KU894721) | 36.10 | Soil |
|  | OTU6 | 31.16 | Hot spring (In this study) |
|  | *Brocadia* *sinica* (BAFN01000001) | 24.53 | Reactor |
|  | *Brocadia* *anammoxidans* (AF375994) | 21.49 | Reactor |
|  | Oil reservoir (HM208774) | 34.31 | Oil reservoir |
|  | Riparian sediment (KP938359) | 36.10 | Riparian sediment |
|  | OTU28 | 36.10 | Hot spring (In this study) |
|  | Oil reservoir (HM208772) | 31.83 | Oil reservoir |
|  | Oil reservoir (HM208773) | 31.83 | Oil reservoir |
|  | Paddy soil (KJ508417) | 37.33 | Paddy soil |
|  | OTU29 | 33.63 | Hot spring (In this study) |
|  | Sea sediment (MH122083) | 38.43 | Sea sedimen |
|  | OTU30 | 39.80 | Hot spring (In this study) |
| *Candidatus* Brocadia | *Brocadia* *sapporoensis* (AM285341) | 26.42 | Reactor |
|  | Freshwater lake sediment (MH969658) | 36.10 | Freshwater lake |
|  | OTU27 | 36.10 | Hot spring (In this study) |

^a^ The optimal growth temperature of each sequence was calculated as Kimura et al., 2006.

**Reference**

Kimura, H., Sugihara, M., Kato, K., Hanada, S. (2006). Selective phylogenetic analysis targeted at 16S rRNA genes of thermophiles and hyperthermophiles in deep-subsurface geothermal environments. *Appl. Environ. Microbiol*. 72, 21-27. doi: 10.1128/AEM.72.1.21–27.2006.
